# Dual inhibition of ErbB1 and ErbB2 in cancer by recombinant human prolidase mutant hPEPD-G278D

**DOI:** 10.18632/oncotarget.9851

**Published:** 2016-06-06

**Authors:** Lu Yang, Yun Li, Arup Bhattacharya, Yuesheng Zhang

**Affiliations:** ^1^ Departments of Pharmacology and Therapeutics, Roswell Park Cancer Institute, Buffalo, NY, USA; ^2^ Urology, Roswell Park Cancer Institute, Buffalo, NY, USA; ^3^ Cancer Prevention and Control, Roswell Park Cancer Institute, Buffalo, NY, USA

**Keywords:** ErbB1, ErbB2, ErbB1 inhibitor, ErbB2 inhibitor, prolidase

## Abstract

ErbB1 and ErbB2 are oncogenic cell surface receptor tyrosine kinases, linked to many forms of human cancer, and are major cancer therapeutic targets. Many lines of evidence indicate that targeting ErbB1 and ErbB2 is an important cancer therapeutic approach. We recently found that a recombinant enzymatically-inactive mutant of human prolidase, i.e., hPEPD-G278D, is an inhibitory ligand of ErbB2 and strongly inhibits ErbB2-overexpressing cells in vitro and in vivo. hPEPD-G278D also binds to ErbB1. Here, we show that hPEPD-G278D binds to ErbB1 with high affinity, initially activating ErbB1 but later silencing it, and that deletion of subdomain 2 in ErbB1 extracellular domain abolishes the binding. The proliferation of ErbB1-overexpressing cells is strongly inhibited by hPEPD-G278D, regardless of ErbB2 expression or cell type, whereas cells lacking ErbB1 and ErbB2 are insensitive to it. In contrast, EGF, another ErbB1 ligand, either stimulates or mildly inhibits cell proliferation. Moreover, hPEPD-G278D treatment of mice bearing ErbB1-overexpressing tumors leads to tumor regression, which is accompanied by down regulation and decreased phosphorylation of ErbB1 and ErbB2 as well as decreased phosphorylation of downstream signaling molecules and activation of apoptosis in the tumor tissues. We conclude that hPEPD-G278D is a dual inhibitor of ErbB1 and ErbB2 and selectively targets cancer cells overexpressing ErbB1 and/or ErbB2. Moreover, our finding that both receptors are silenced in cancer cells by hPEPD-G278D highlights an unusual consequence of ligand-receptor interaction.

## INTRODUCTION

ErbB1 and ErbB2 are members of the ErbB family of cell surface receptor tyrosine kinases, which also include ErbB3 and ErbB4, and are important therapeutic targets in many forms of cancer [1, 2]. Two main classes of agents have been developed to target ErbB1 and ErbB2, including monoclonal antibodies that bind to the extracellular domain (ECD) of the receptors (e.g., cetuximab and trastuzumab) and tyrosine kinase inhibitors that bind to their intracellular kinase domains (e.g., gefitinib and lapatinib). Notably, lapatinib targets both ErbB1 and ErbB2 [3]. Many lines of evidence indicate that targeting both ErbB1 and ErbB2 is an important approach in cancer treatment. For example, while ErbB2 overexpression in breast cancer is a strong predictor of poor disease prognosis [4, 5], ErbB2 overexpression accompanied by ErbB1 expression is associated with worse prognosis [6, 7]. Conversely, increased ErbB2 expression causes cancer resistance to ErbB1-directed therapies, e.g., cetuximab that specifically targets ErbB1 [8]. However, lapatinib is crippled by primary and acquired resistance [9]. Although it is approved for use in combination with capecitabine or letrozole for treating ErbB2-positive breast cancer, adjuvant lapatinib as a single agent for women with early-stage ErbB2-positive breast cancer shows marginal efficacy [10]. Moreover, adjuvant or neoadjuvant treatment of ErbB2-positive breast cancer with lapatinib and ErbB2-specific trastuzumab does not significantly improve disease-free survival, compared to trastuzumab alone [11, 12]. New agents that are capable of dual inhibition of ErbB1 and ErbB2 are greatly needed.

We recently found that human prolidase, also known as peptidase D (PEPD), is a high affinity ligand of human ErbB2 (Kd = 7.3 nM) and binds to subdomain 3 of ErbB2 ECD [13]. This was significant, because no natural ErbB2 ligand had been previously found, while ligands for all other ErbBs had been identified [1]. The physiological relevance of PEPD as an ErbB2 ligand remains unclear, but we have shown that in ErbB2-overexpressing cells, recombinant human PEPD (hPEPD) is an inhibitory ligand of ErbB2; it disrupts oncogenic signaling of ErbB2 (disrupting both homodimeric and heterodimeric signaling units), down regulates its expression via internalization and degradation, and inhibits cell growth [13, 14]. Moreover, hPEPD treatment of mice bearing ErbB2-overexpressing tumors resulted in strong inhibition of tumor growth, which was accompanied by marked down regulation and decreased phosphorylation of ErbB2 as well as decreased phosphorylation of downstream signaling molecules, such as SRC, AKT, extracellular signal-regulated kinase (ERK) and signal transducer and activator of transcription 3 (STAT3) in the tumor tissues [14]. In contrast, hPEPD was ineffective against cells and tumors expressing very low ErbB2 and no other ErbBs [13, 14]. hPEPD is a homodimeric depeptidase (493 amino acids [aa] per subunit), involved in collagen metabolism [15, 16], but its enzymatic function plays no role in hPEPD interaction with ErbB2 [13]. In fact, an enzymatically inactive mutant of hPEPD, i.e., hPEPD-G278D (change from glycine to aspartic acid at position 278), was significantly more effective than hPEPD in inhibiting ErbB2-overexpressing tumors in mice, and hPEPD, but not hPEPD-G278D, stimulated prosurvival hypoxia inducible factor 1α in the tumor tissues [14]. Given that hPEPD-G278D is a human protein and is unlikely to interfere with the enzymatic function of endogenous PEPD in normal cells, it is a highly promising agent for combating ErbB2-overexpressing cancer. Notably, we detected no adverse effects in mice treated with hPEPD-G278D at the dose regimen that strongly inhibited tumor growth [14].

Neither hPEPD-G278D nor hPEPD binds to ErbB3 or ErbB4 [13], but both of them also bind to ErbB1 [14, 17]. Interestingly, hPEPD and its mutant do not carry an epidermal growth factor (EGF) domain which exists in all other ErbB1 ligands and is believed to be important for ligand-receptor binding [18]. Moreover, each subunit of the homodimeric hPEPD-G278D or hPEPD binds to one monomer of ErbB1, thereby forming a tetramer [14], resembling their binding to ErbB2 [13]. In contrast, all other ligands bind to ErbB1 at one molecule of ligand per monomer of receptor [1, 19]. hPEPD-G278D and hPEPD, therefore, represent a new class of ErbB1 ligands and also show that an EGF domain is not a prerequisite for an ErbB1 ligand. In cultured cells, both proteins first stimulate and then down regulate ErbB1 at low nM concentrations [14, 17]. However, when hPEPD was measured against human ErbB1 ECD fused to the Fc fragment of human IgG1, its binding affinity was unusually low (Kd = ~5 μM) [17]. In the present study, we have addressed this discrepancy by measuring the binding affinities of both hPEPD-G278D and hPEPD towards full-length human ErbB1. We have also identified the location in ErbB1 ECD to which hPEPD-G278D may bind. Most importantly, we have addressed the question of whether hPEPD-G278D interaction with ErbB1 leads to stimulation or inhibition of cell proliferation and tumor growth. Our data show that hPEPD-G278D is a potent dual inhibitor of ErbB1 and ErbB2, which is mechanistically distinct from lapatinib.

## RESULTS

### hPEPD and hPEPD-G278D bind to ErbB1 with high affinity

**Figure 1:**
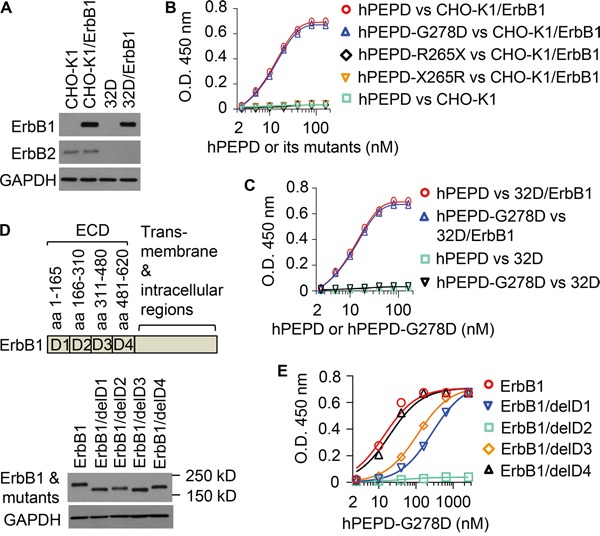
Binding of hPEPD and its mutants to human ErbB1 **A.** Levels of ErbB1 and ErbB2 in various cell lysates, measured by IB. **B, C.** Binding of hPEPD and its mutants to ErbB1 in CHO-K1/ErbB1 cell lysates or 32D/ErbB1 cell lysates, measured by ELISA, using CHO-K1 cell lysates and 32D cell lysates as controls. **D, E.** ErbB1 deletion mutants and their expression levels in CHO-K1 cells after transient gene transfection, measured by IB, and hPEPD-G278D binding to ErbB1 and its mutants in the cell lysates, measured by ELISA. Each value in (B, C, E) is mean ± SD (n=3).

We previously showed that hPEPD binds to ErbB1 ECD [17]. Here, the binding affinity of hPEPD and its mutants to full length ErbB1 was measured. Chinese hamster ovary CHO-K1 cells express very low ErbB2 and no other ErbBs [13]. Human ErbB1 was stably expressed in CHO-K1 cells (CHO-K1/ErbB1) (Figure 1A). The lysates of these cells were used to measure the binding of hPEPD and its mutants towards ErbB1 by the enzyme-linked immunosorbent assay (ELISA). hPEPD showed little non-specific binding (CHO-K1 cell lysates) but bound to ErbB1 (CHO-K1/ErbB1 cell lysates) in a dose-dependent manner with a dissociation constant (Kd) of 17.7 nM (95% confidence interval = 12.7-22.7 nM) (Figure 1B). The binding affinity of hPEPD-G278D towards ErbB1 is seemingly identical to that of hPEPD, but two deletion mutants, i.e., hPEPD-R265X (deletion of aa # 266-493) and hPEPD-X265R (deletion of aa # 1-264) (Supplementary Figure 1), failed to bind to ErbB1 (Figure 1B). This leaves the ErbB1-binding site in hPEPD undefined, but neither deletion mutant can form homodimer [13], which probably renders it unable to bind to ErbB1. We ruled out a cell line-specific effect or involvement of ErbB2 in ErbB1 binding to hPEPD and hPEPD-G278D by repeating the binding experiment using the lysates of murine myeloid 32D cells (no expression of any ErbBs) and 32D/ErbB1 cells which stably express human ErbB1 [20]. Levels of ErbB1 and ErbB2 in these cells are shown in Figure 1A. Neither hPEPD nor hPEPD-G278D showed significant non-specific binding (32D cell lysates), but each showed dose-dependent and seemingly identical binding towards ErbB1 (Kd = 17.3-17.6 nM, 95% confidence interval =12.7-22.4 nM) (Figure 1C). The Kd values described above are more than 300-fold lower than that measured using ErbB1/ECD-Fc (the ECD is fused to the Fc fragment of human IgG1) [17], but are consistent with our repeated observations that both hPEPD and hPEPD-G278D stimulate and then down regulate ErbB1 in cultured cells at low nM concentrations [14, 17]. Because each subunit of the homodimeric hPEPD or hPEPD-G278D binds to one monomer of ErbB1, giving rise to a tetramer [14], the Fc fragment in the ErbB1/ECD-Fc chimera may interfere with binding of hPEPD and its mutant to the ECD. However, the possibility that certain factor(s) in cell lysates facilitates binding of hPEPD and its mutant to ErbB1 cannot be ruled out.

### Deletion of subdomain 2 in ErbB1 ECD abolishes hPEPD-G278D binding

We next tried to determine the location in ErbB1 to which hPEPD-G278D may bind. Human ErbB1 mutants with deletion of one of the four ECD subdomains [21] were generated by site-directed mutagenesis and expressed in CHO-K1 cells (Figure 1D). hPEPD-G278D binding to ErbB1 and its mutants in cell lysates were compared by ELISA (Figure 1E). Deletion of subdomain 1 or 3 reduced hPEPD-G278D binding affinity by 19.2-fold and 7.4-fold, respectively, on the basis of Kd value change, but full binding was achieved at high hPEPD-G278D concentration, whereas deletion of subdomain 2 totally abolished hPEPD-G278D binding. Deletion of subdomain 4 had little effect on hPEPD-G278D binding, as its binding affinity deceased by only 1.4-fold (Figure 1E). These results indicate that hPEPD-G278D may bind to ErbB1 ECD subdomain 2 but that subdomains 1 and 3 may play a significant role in facilitating the binding. However, the possibility that deletion of a specific subdomain disrupts the orientation or conformation of other subdomains, thereby abolishing or diminishing hPEPD-G278D binding to ErbB1, cannot be ruled out. Notably, we previously showed that hPEPD binds to subdomain 3 of human ErbB2 ECD [13], while EGF interacts with both subdomains 1 and 3 in ErbB1 ECD [22].

### Overexpression of ErbB1 sensitizes cells to inhibition by hPEPD-G278D

**Figure 2:**
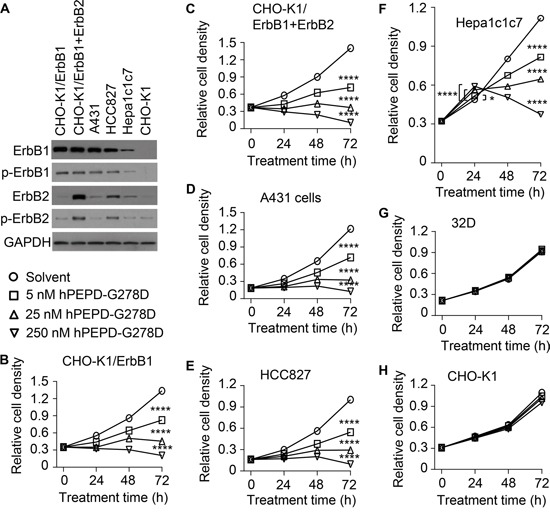
Inhibition of cell proliferation by hPEPD-G278D **A.** Expression of ErbB1 and ErbB2 as well as p-Y1173-ErbB1 and p-Y1221/1222-ErbB2 in cell lines prior to hPEPD-G278D treatment, measured by IB. **B-H.** Cells were treated with solvent or hPEPD-G278D as indicated, followed by measurement of cell density by the MTT assay. Each value is mean ± SD (n=3). *P<0.05, ****P<0.0001.

We previously showed that ErbB2 overexpression sensitizes cells to inhibition by hPEPD and hPEPD-G278D [13, 14]. Here, hPEPD-G278D was evaluated in a panel of 7 cell lines with or without expression of ErbB1 and/or ErbB2. CHO-K1 cells express very low ErbB2 and no other ErbBs. CHO-K1/ErbB1 cells overexpress ErbB1, and CHO-K1/ErbB1+ErbB2 cells overexpress both ErbB1 and ErbB2. Human skin cancer A431 cells, which are widely used in ErbB1 research, overexpress ErbB1 and also express a low level of ErbB2. Human lung cancer HCC827 cells overexpress ErbB1 with an activating mutation in its tyrosine kinase domain [23, 24] and also have significant expression of ErbB2. Murine hepatoma Hepa1c1c7 cells express moderate to low level of ErbB1 and ErbB2, whereas 32D cells have no expression of any ErbBs. The relative expression levels of ErbB1 and ErbB2 in these cell lines are shown in Figure 2A as well as Figure 1A. Cells were treated with solvent or hPEPD-G278D at 5, 25 and 250 nM for 24, 48 and 72 h, followed by measurement of cell proliferation. hPEPD-G278D inhibited the proliferation of CHO-K1/ErbB1 cells, CHO-K1/ErbB1+ErbB2 cells, A431 cells and HCC827 cells in a dose- and time-dependent manner; after 72 h treatment with 250 nM hPEPD-G278D, cell proliferation was inhibited 84.7-92.5% (Figure 2B-2E). Interestingly, hPEPD-G278D first stimulated the proliferation of Hepa1c1c7 cells (up to 21.4% after 24 h treatment) but then inhibited their proliferation (up to 66.7% inhibition after 72 h treatment) (Figure 2F). Treatment of Hepa1c1c7 cells with hPEPD for 24 h also significantly increases their proliferation [17]. 32D cells were insensitive to hPEPD-G278D (Figure 2G), whereas the proliferation of CHO-K1 cells was only marginally inhibited by it (Figure 2H), likely due to the presence of a small amount of ErbB2 in these cells.

### hPEPD-G278D silences ErbB1, ErbB2 and their downstream signaling molecules

**Figure 3:**
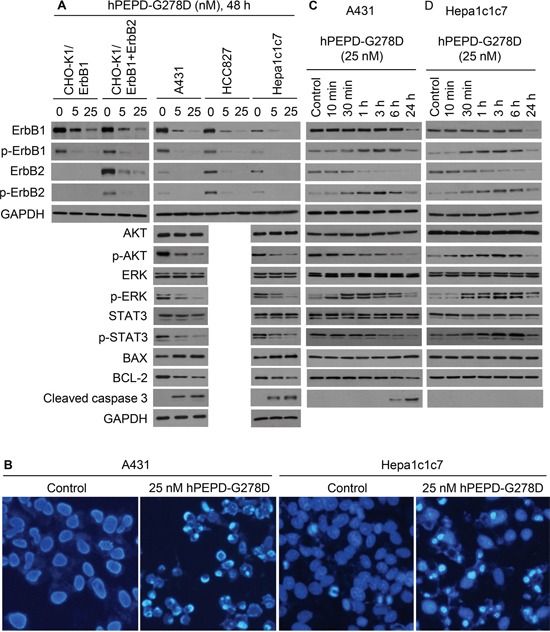
Effect of hPEPD-G278D on ErbB1, ErbB2, their downstream signaling and regulatory molecules, and cell morphology **A-D.** Cells were treated by solvent or hPEPD-G278D as indicated, followed by IB or DAPI staining. Measurement of protein phosphorylation by IB includes p-Y1173-ErbB1, p-Y1221/1222-ErbB2, p-S473-AKT, p-T202/Y204-ERK and p-Y705-STAT3. Cells were stained by DAPI after treatment with solvent or hPEPD-G278D for 48 h (10X magnification).

Treatment with hPEPD-G278D at 5 and 25 nM for 48 h caused dose-dependent down regulation and decreased phosphorylation (measuring representative phosphorylation sites) of both ErbB1 and ErbB2 in all cell lines examined, including CHO-K1/ErbB1 cells, CHO-K1/ErbB1+ErbB2 cells, A431 cells, HCC827 cells and Hepa1c1c7 cells (Figure 3A). Notably, the Hepa1c1c7 result also shows that hPEPD-G278D targets murine ErbB1 and ErbB2. We next examined several important signaling or regulatory proteins that are downstream of ErbB1 and ErbB2 in A431 cells and Hepa1c1c7 cells, including AKT, ERK, STAT3, B-cell lymphoma 2 (BCL-2), BCL-2-associated X protein (BAX), and caspase-3. hPEPD-G278D had no effect on the expression of AKT, ERK and STAT3 but caused dose-dependent decrease in phosphorylation of these proteins in both cell lines (Figure 3A). The phosphorylation sites measured, including p-S473-AKT, p-T202/Y204-ERK and p-Y705-STAT3, are critical for the functions of the proteins [25–27]. hPEPD-G278D also dose dependently up regulated BAX, down regulated BCL-2, and activated caspase-3 (Figure 3A), indicating activation of apoptosis. Indeed, 4,6-diamidino-2-phenylindole (DPAI) staining of A431 cells and Hepa1c1c7 cells after treatment with hPEPD-G278D at 25 nM for 48 h showed marked increase in apoptosis in both cell lines (chromatin condensation and nuclear fragmentation into dense granular particles known as apoptotic bodies) (Figure 3B).

To assess changes induced by hPEPD-G278D at earlier time points, A431 cells and Hepa1c1c7 cells were treated with the agent at 25 nM for 10 min to 24 h. In both cell lines treated by hPEPD-G278D, phosphorylation of ErbB1 and ErbB2 began to increase at 10-30 min, peaked at 3 h but largely returned to basal level at 24 h; ErbB1 expression did not change in the first 6 h but decreased at 24 h, whereas ErbB2 expression began to decrease as early as 1 h (Figure 3C, 3D; Supplementary Figure 2 shows no effect of solvent on ErbB1 and ErbB2 as well as their phosphorylation status). Similar changes were observed in other cell lines [13, 14, 17], apparently resulting from ligand-receptor binding, receptor dimerization, and receptor degradation via internalization. The relatively slow onset of increased phosphorylation of ErbB1 and ErbB2 in hPEPD-G278D-treated cells is consistent with the time line of homodimerization of ErbB1 or ErbB2 induced by hPEPD-G278D or hPEPD [13, 14]. The marked difference between ErbB1 and ErbB2 in terms of the pace of their down regulation by hPEPD-G278D may be related to their internalization mechanism. Clathrin-mediated internalization of ErbB1 recycles it to cell surface, whereas clathrin-independent internalization of ErbB1 commits it to degradation [28]. ErbB2 was shown to undergo clathrin-independent internalization and degradation [29]. Interestingly, while hPEPD-G278D also transiently stimulated the phosphorylation of AKT, ERK and STAT3 in Hepa1c1c7 cells in a time line that matches that of phosphorylation of ErbB1 and ErbB2, indicating rapid signal transmission from the receptors (Figure 3D), only ERK showed such change in A431 cells (Figure 3C). hPEPD-G278D caused rapid decrease in phosphorylation of both AKT and STAT3 in A431 cells (Figure 3C). The reason for the change in AKT and STAT3 in A431 cells is not entirely clear, but we previously showed in other cell lines that hPEPD rapidly silences SRC signaling by disrupting its association with ErbB2 [13], which may silence AKT and STAT3. The different response of AKT and STAT3 to hPEPD-G278D in A431 cells and Hepa1c1c7 cells provides a clue as to why hPEPD-G278D treatment for 24 h stimulates the proliferation of Hepa1c1c7 cells but inhibited the proliferation of A431 cells (Figure 2D, 2F).

### Cells respond to hPEPD-G278D and EGF differently

**Figure 4:**
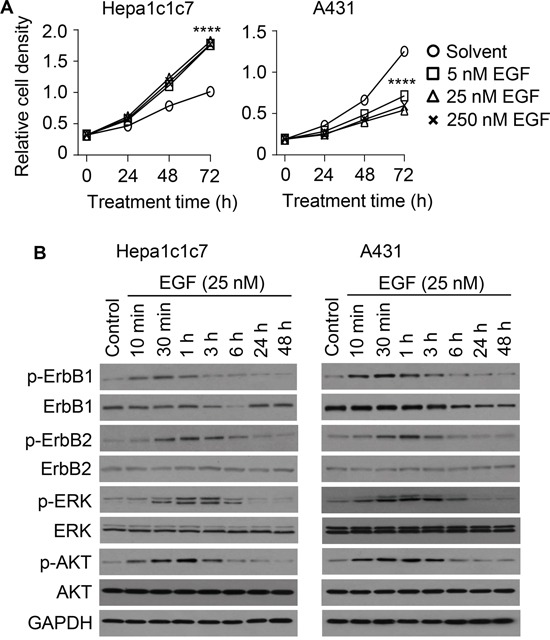
Effect of EGF on cell proliferation, ErbB1, ErbB2 and their downstream signaling molecules **A.** Cells were treated by solvent or EGF as indicated, followed by measurement of cell density by the MTT assay. Each value is mean ± SD (n=3). ****P<0.0001. **B.** Cells were untreated or treated by EGF as indicated, from which cell lysates were prepared and analyzed by IB. Measurement of protein phosphorylation includes p-Y1173-ErbB1, p-Y1221/1222-ErbB2, p-T202/Y204-ERK and p-S473-AKT.

We also evaluated EGF in Hepa1c1c7 cells and A431 cells, and for comparison with hPEPD-G278D, the cells were treated with EGF at 5, 25 and 250 nM for 24, 48 and 72 h. EGF stimulated Hepa1c1c7 cell proliferation in a time-dependent manner but achieved maximal effect at 5 nM (Figure 4A). In contrast, EGF inhibited A431 cell proliferation (Figure 4A), but the inhibition was relatively modest, compared to that of hPEPD-G278D (Figure 2D), and plateaued at 5-25 nM with maximal inhibition of 56.7% after 72 h treatment (Figure 4A).

We next treated A431 cells and Hepa1c1c7 cells with EGF at 25 nM for 10 min to 48 h and then analyzed ErbB1, ErbB2 and representative downstream signaling molecules. EGF caused rapid but transient phosphorylation of both ErbB1 and ErbB2 in both cell lines (peaked at 30 min and returned to basal level at 3-6 h) (Figure 4B). EGF apparently induces ErbB2 phosphorylation by stimulating ErbB1-ErbB2 heterodimerization, as EGF is not an ErbB2 ligand and has no effect on ErbB2 in cells without ErbB1 [13]. Notably, hPEPD-G278D disrupts ErbB1-ErbB2 association [14]. ErbB1 level began to decrease at 3-6 h of EGF treatment in both cell lines, and returned to basal level at 24 h in Hepa1c1c7 cells but continued to decrease gradually for at least 48 h in A431 cells (Figure 4B). However, EGF-induced ErbB1 decrease in A431 cells is less than that induced by hPEPD-G278D (Figure 3A). EGF did not cause a change in ErbB2 expression in either cell line (Figure 4B). Notably, EGF-induced ErbB1-ErbB2 heterodimers tend not to be internalized [30]. Both Hepa1c1c7 cells and A431 cells also show transient increase in phosphorylation of AKT and ERK after EGF treatment, in a time course that matches that of phosphorylation of ErbB1 and ErbB2 (Figure 4B). Collectively, the molecular changes induced by EGF in the two cell lines seem similar, except for ErbB1 expression level; however, to what extent the difference in ErbB1 expression change in the two cell lines may account for the differential effect of EGF on their proliferation remains unknown. Barnes suggested that EGF may induce A431 cell differentiation [31]. However, several changes at the molecular level clearly set EGF apart from hPEPD-G278D, including the inability of EGF to silence ErbB2 and to cause sustained and profound depletion of ErbB1.

### hPEPD-G278D strongly inhibits ErbB1-overexpressing tumors in vivo

Despite inhibition of A431 cell proliferation by EGF in vitro, a previous study showed that administration of EGF (approximately 1.5 mg/kg body weight, 3 times weekly) has no effect on the growth of subcutaneous A431 tumors in athymic mice [32]. It was therefore of interest to test hPEPD-G278D against A431 tumors in vivo. Moreover, A431 cells overexpress ErbB1 but express a low level of ErbB2 (Figure 2A), making it a suitable model for evaluation of hPEPD-G278D against ErbB1-driven oncogenesis.

**Figure 5:**
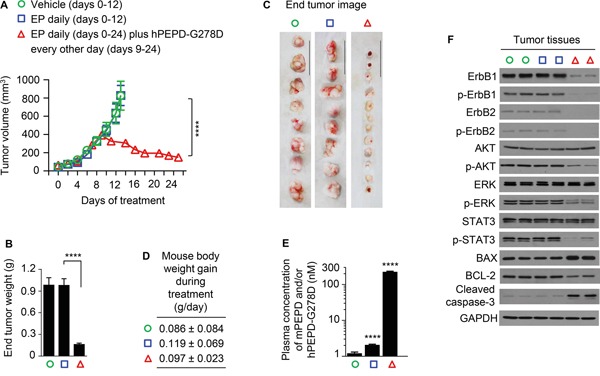
Inhibition of ErbB1-overexpressing tumors by hPEPD-G278D A431 cells were inoculated subcutaneously to athymic mice; once tumor size reached 40-50 mm^3^, the mice were treated by vehicle or EP (0.5 mg/kg) i.p. daily. Another group of mice were also treated with EP (0.5 mg/) i.p. daily, but nine days later, while continuing EP treatment, the mice also began treatment with hPEPD-G278D (4 mg/kg) i.p. every other day. The treatment period differs among different experimental groups due to difference in tumor growth, but the mice were killed 24 h after the final treatment in each group. **A.** Tumor size (mean ± SEM) upon treatment with vehicle (n=8), EP only (n=8), or EP plus hPEPD-G278D (n=12). **B, C.** Tumor weight (mean ± SD) and image of individual tumors (scale bar: 3 cm) at 24 h following final treatment. **D.** Mouse body weight gain during experimental treatment (mean ± SD). **E.** Plasma levels of mPEPD and/or hPEPD-G278D at 24 h following final treatment (mean ± SD), measured by ELISA. **F.** Molecular changes induced by hPEPD-G278D in the tumors, measured by IB; each lane represents one tumor. Measurement of protein phosphorylation includes p-Y1173-ErbB1, p-Y1221/1222-ErbB2, p-S473-AKT, p-T202/Y204-ERK, and p-Y705-STAT3. ****P<0.0001.

hPEPD-G278D was evaluated in combination with enoxaparin (EP), a low molecular weight heparin and a clinically used anticoagulant. We previously found that only low nM plasma levels of hPEPD-G278D or hPEPD could be achieved in mice after injecting them, but EP markedly elevated their plasma levels [14]. We subsequently found that EP inhibits their degradation by a plasma proteolysis pathway composed of coagulation proteases [33]. Although EP itself shows no effect on ErbB2 signaling and tumor growth in several mouse tumor models [14], it was not previously evaluated in A431 tumors in vivo. Therefore, our experiment was designed to have three experimental groups: a vehicle group, an EP only group, and a group of hPEPD-G278D in combination with EP. A431 cells were inoculated subcutaneously to athymic mice; once tumor size reached 40-50 mm^3^, the mice were treated with vehicle or EP at 0.5 mg/kg daily by intraperitoneal injection (i.p.). We previously showed that daily EP at 0.5 mg/kg is adequate for inhibition of hPEPD-G278D degradation in mice [14]. In the hPEPD-G278D plus EP group, the mice were first treated with EP daily until their tumors reached approximately 400 mm^3^ (9 days of EP treatment) and then treated with daily EP plus hPEPD-G278D which was administered to the mice at 4 mg/kg i.p. every other day. The hPEPD-G278D dose regimen was selected based on our recent data showing that hPEPD-G278D at 2 mg/kg thrice weekly i.p., while achieving strong antitumor activity in a mouse model of human breast cancer, generated plasma drug concentration of only about 130 nM [14]. The A431 tumors grew exceedingly fast, but EP had no effect on tumor growth (Figure 5A). Mice treated with either vehicle or EP alone were killed when their tumors reached beyond 800 mm^3^, as some of the tumors began to show sign of potential necrosis. However, tumor regression occurred even after the first dose of hPEPD-G278D and became more pronounced as the treatment continued (Figure 5A). Mice treated with hPEPD-G278D plus EP were killed when the size of their tumors decreased to approximately 150 mm^3^, when 58.3% (7 out of 12) of the tumors became necrotic. The exact reason for the necrosis of the shrinking tumors is not known, but it is apparently treatment-related. Even though the mice treated with EP plus hPEPD-G278D were killed 12 days later than those treated with vehicle or EP alone, their tumors on average were 85% smaller than those in the latter groups (Figure 5B-5C). All mice were killed 24 h after the final treatment. No adverse effects were detected in mice treated by EP or EP plus hPEPD-G278D, and neither agent had a significant effect on body weight gain of the mice (Figure 5D). Plasma levels of endogenous mouse PEPD (mPEPD) or mPEPD plus hPEPD-G278D were measured at 24 h after the final treatment. Average plasma level of mPEPD was 1.2 nM in the vehicle-treated mice and 2.1 nM in the mice treated by EP only, whereas average plasma level of mPEPD plus hPEPD-G278D in the mice treated by EP plus hPEPD-G278D was 227.0 nM (Figure 5E). Thus, EP only slightly increased plasma mPEPD level but enables hPEPD-G278D to reach high concentration in the plasma, as shown previously [14].

EP had no effect on any of the signaling molecules measured in the tumor tissues (Figure 5F). However, hPEPD-G278D caused down regulation and decreased phosphorylation of both ErbB1 and ErbB2 as well as decreased phosphorylation of all three downstream signaling molecules that were measured, including AKT, ERK, and STAT3. hPEPD-G278D also up regulated BAX, down regulated BCL-2 and activated caspase-3 in the tumor tissues (Figure 5F), which is consistent with marked tumor regression upon hPEPD-G278D treatment. The impact of hPEPD-G278D on the signaling molecules in A431 tumors is similar to that in cultured A431 cells (Figure 3A). Our results show that hPEPD-G278D effectively targets both ErbB1 and ErbB2 in ErbB1-overexpressing human cancer cells in vivo. hPEPD-G278D also targets both ErbB1 and ErbB2 in ErbB2-overexpressing human breast BT-474 tumors in vivo [14].

## DISCUSSION

We previously showed that hPEPD-G278D is highly effective against ErbB2-overexpressing cells in vitro and in vivo [13, 14]. Our present study shows that cells that overexpress ErbB1, regardless of ErbB2 expression, are also strongly inhibited by this agent in vitro and in vivo, whereas cells that do not express or express very low levels of these receptors are insensitive to it. Besides directly binding and silencing ErbB1 and ErbB2, we previously showed that hPEPD-G278D also disrupts ErbB2-ErbB3 heterodimer interaction, regardless of stimulation by ErbB3 ligand neuregulin 1, and disrupts ErbB1-ErbB2 heterodimer interaction, regardless of stimulation by EGF [14]. However, because hPEPD-G278D binds to both ErbB1 and ErbB2, it was previously puzzling that it disrupts ErbB1-ErbB2 heterodimerization. We have now shown that deletion of subdomain 2 in ErbB1 ECD abolishes hPEPD-G278D binding to ErbB1, whereas hPEPD-G278D binds to subdomain 3 of ErbB2 ECD [13]. This likely prevents the two subunits of hPEPD-G278D homodimer from binding to ErbB1 and ErbB2 simultaneously on cell surface. Collectively, our data show that hPEPD-G278D is a strong dual inhibitor of ErbB1 and ErbB2 in cancer.

Lapatinib is a clinically approved dual inhibitor of ErbB1 and ErbB2 in cancer, but its mechanism differs from that of hPEPD-G278D. Lapatinib must enter cells to bind and inhibit the tyrosine kinase domain in the cytoplasmic section of the receptors, whereas hPEPD-G278D binds to the extracellular domains of ErbB1 and ErbB2, disrupting their oncogenic signaling and inducing their degradation via endocytosis. hPEPD-G278D initially stimulates tyrosine phosphorylation of both ErbB1 and ErbB2 due to receptor dimerization, which is transmitted to certain downstream signaling molecules, but lapatinib may counter such effect of hPEPD-G278D. Therefore, experiments to investigate whether the two agents may complement each other for inhibition of ErbB1 and ErbB2 in cancer cell are warranted. hPEPD-G278D may also be a candidate for combination with other inhibitors of ErbB1 and ErbB2. For example, it may be important to investigate whether hPEPD-G278D may synergize with trastuzumab to target ErbB2-overexpressing human breast cancer. Trastuzumab works in part by eliciting antibody-dependent cell mediated cytotoxicity [34, 35], whereas hPEPD-G278D lacks such activity. Yet, hPEPD-G278D targets both ErbB1 and ErbB2, while trastuzumab binds only to ErbB2 and fails to abolish ErbB2 phosphorylation [36]. The strong antitumor activity of hPEPD-G278D in our preclinical studies also raises the possibility that hPEPD-G278D may combat cancer resistance to lapatinib, trastuzumab and other agents. For example, increased ErbB2 expression causes resistance of ErbB1-overexpressing cancer cells to cetuximab which specifically targets ErbB1 [8]. hPEPD-G278D may inhibit these cancers by silencing both ErbB1 and ErbB2.

Overexpression of ErbB1 and/or ErbB2 occurs in many types of human cancer, such as breast cancer, colon cancer, head and neck cancer, and lung cancer. Evaluation of hPEPD-G278D in relevant experimental models seems warranted. Indeed, our results indicate that the ability of hPEPD-G278D to inhibit both ErbB1 and ErbB2 is not limited to a specific cell type.

## MATERIALS AND METHODS

### Biochemicals

hPEPD, hPEPD-G278D, hPEPD-R265X and hPEPD-X265R were produced in *E.coli* as previously described [13, 17]. EP and human EGF (236-EG-200) were purchased from Fresenius Kabi and R&D Systems, respectively. The following antibodies were used in the study: anti-PEPD (Abcam, ab86507) which detects hPEPD, hPEPD-G278D and mPEPD, anti-ErbB1 (Cell Signaling, 2232), anti-p-ErbB1 (Y1173) (Cell Signaling, 4407), anti-ErbB2 (Cell Signaling, 2165), anti-p-ErbB2 (Y1221/1222) (Cell Signaling, 2243), anti-AKT (Cell Signaling, 4691), anti-p-AKT (Cell Signaling, 4060), anti-ERK (Cell Signaling, 9102), anti-p-ERK (Cell Signaling, 9101), anti-STAT3 (Cell Signaling, 4904), anti-p-STAT3 (Cell Signaling, 9145), anti-cleaved caspase-3 (Cell Signaling, 9661), anti-BCL-2 (Cell Signaling, 2870), anti-BAX (Cell Signaling, 2772), anti-glyceraldehyde 3-phosphate dehydrogenase (GAPDH) (Millipore, MAB374), and biotin-conjugated anti-6XHistidines (His)-tag (Bethyl, A190-113B). Horseradish peroxidase (HRP)-conjugated streptavidin (N100) was purchased from Thermo Scientific. A goat anti-rabbit IgG-HRP was purchased from Jackson ImmunoResearch (111-035-003).

### Plasmid construction

To generate an expression vector of human ErbB1 with puromycin selection marker (pCMV6-A-ERBB1-Puro), pCMV6-XL5-ERBB1 [14] was used as a template to amplify ERBB1 by PCR using SgfI-forward primer and MIuI-reverse primer. The amplified PCR product was digested by SgfI and MIuI (Thermo Scientific) and subcloned into pCMV6-A-Puro (Origene). pCMV6-XL5-ERBB1 was also used to generate mutants lacking ErbB1 ECD subdomain 1 (aa #1-165), 2 (aa #166-310), 3 (aa #311-480) or 4 (aa #481-620), using QuikChange Lightning Site-Directed Mutagenesis Kit (Agilent Technologies). All constructs were confirmed by DNA sequence analysis. All primer sequences are provided in Supplementary Table 1.

### Gene transfection

Cells were grown in 6-well plates and transfected with a specific plasmid at 1-2 μg DNA per well, using FuGENE HD (Promega).

### Cell lines and cell culture

CHO-K1 cells, A431 cells, HCC827 cells were from American Type Culture Collection. Hepa1c1c7 cells, 32D cells, and 32D/ErbB1 cells have been previously described [17]. CHO-K1/ErbB1 cells and CHO-K1/ErbB1+ErbB2 cells were generated by transfecting CHO-K1 cells and CHO-K1/ErbB2 cells [13] with pCMV6-A-ERBB1-Puro and selection under puromycin. CHO-K1 cells and their derivatives were cultured in F-12K medium (Gibco) supplemented with 10% fetal bovine serum (FBS, Gibco). A431 cells were cultured in high glucose Dulbecco's modified Eagle's medium supplemented with 10% FBS. HCC827 cells were cultured in RPMI-1640 medium supplemented with 1% 4-(2-hydroxyethyl)-1-piperazineethanesulfonic acid, 1% sodium pyruvate, 0.6% glucose and 10% FBS. Hepa1c1c7 cells, 32D cells and 32D/ErbB1 cells were cultured as previously described [17]. All cell lines were cultured in humidified incubators at 37°C with 5% CO_2_.

### Immunoblotting (IB)

Sample preparation and assay protocol are the same as previously published [13, 17]. GAPDH was used as a loading control.

### Measurement of binding of hPEPD and its mutants to ErbB1 and its mutants by ELISA

ELISA plate wells were coated with an ErbB1 antibody (binding to the cytoplasmic tail of ErbB1) by incubating with 100 μl/well of the antibody (10 μg/ml) overnight at 4°C. After washing the wells three times with phosphate-buffered saline with tween 20 (PBST), residual protein binding sites in the wells were blocked by incubation for 2 h at room temperature (RT) with 300 μl/well of 1% bovine serum albumin in phosphate-buffered saline (PBS). Following addition of 60 μl of serially diluted hPEPD, hPEPD-G278D or other mutants to each well, 60 μl of cell lysates containing 25 μg of total protein were added to each well and incubated at 37°C for 2 h. After three washes with PBST, 100 μl of a biotin-conjugated anti-His antibody (1:10,000 dilution; note that hPEPD and hPEPD-G278D as well as other mutants are His-tagged at their carboxyl termini) was added to each well and incubated for 2 h at RT. After another round of washing with PBST, 100 μl of streptavidin-conjugated HRP (1:10,000 dilution) was added to each well and incubated for 45 min at RT. After further washing with PBST, 100 μl/well of 1x substrate solution (3,3′,5,5′-tetramethylbenzedine) was added, and after adequate color development, 100 μl/well of stop solution (1 N H_2_SO_4_) was added, followed by absorbance reading at 450 nm. In the experiment comparing hPEPD-G278D binding to wild-type ErbB1 and its deletion mutants, an equal amount of ErbB1 protein and its mutants was used. CHO-K1 cells were transfected with a specific plasmid for 24 h; cell lysates were then prepared and analyzed by IB, followed by densitometry measurement of the specific protein bands and normalization by a loading control (GAPDH), in order to calculate the amount of lysates that deliver the same amount of each protein (25 μg of total protein/sample were used for lysates carrying the wild-type ErbB1). Cell lysates used in the assay were prepared as follows: Cells were harvested from culture by trypsin treatment and centrifugation, washed twice with PBS, lysed in M-PER buffer (Thermo Scientific) supplemented with 2 mM phenylmethanesulfonyl fluoride and a proteinase inhibitor mix (Roche Applied Science); the mixture was sonicated to enhance cell lysis and cleared of debris by centrifugation at 13,000 x g for 10 min at 4°C. Protein concentrations of all samples were measured by the Bicinchoninic Acid Assay Kit (Pierce).

### Methylthiazolyldiphenyl-tetrazolium bromide (MTT) cell proliferation assay

Cells were grown in 96-well plates (each well was seeded with 500 CHO-K1 cells, 500 CHO-K1/ErbB1, 500 CHO-K1/ErbB1+ErbB2 cells, 500 Hepa1c1c7 cells, 2,000 A431 cells or 2,000 HCC827 cells per well with 150 μl medium) overnight and then treated with solvent, hPEPD-G278D or EGF in 200 μl medium per well for 24, 48 and 72 h, respectively, followed by incubation with MTT (9.2 mM in medium) at 37°C for 3 h. The cells were then washed with PBS and mixed with dimethyl sulfoxide (150 μl per well), and cell density was determined by measuring the reduction of MTT to formazan spectroscopically at 570 nm. 32D cells grew in suspension; 2,000 cells were treated with solvent or hPEPD-G278D in 200 μl medium per well for 24, 48 and 72 h, respectively, followed by incubation with MTT as described above. The cells were then washed with PBS with aid of centrifugation, mixed with dimethyl sulfoxide and measured for cell density as described above.

### DAPI staining

Cells were cultured in 48-well plates (2000 Hepa1c1c7 cells or 6000 A431 cells per well with 0.4 ml medium) with or without hPEPD-G278D treatment (25 nM, 48 h). The cells were then washed with PBS twice, fixed in ice-cold methanol for 15 min, washed again with PBS twice, and incubated with DAPI (1 μg/ml PBS, 0.2 ml/well) for 15 min in the dark. After washing with PBS, the cells were examined using a fluorescence microscope (Axiovert 40 CFL, Carl Zeiss). Images were taken using A-Plan 10 × 0.25 objective lenses (Carl Zeiss) and a Flex Camera (Spot) using the Spot advanced acquisition software.

### Tumor xenograft study

The experimental protocol was approved by the Institutional Animal Care and Use Committee (IACUC) at Roswell Park Cancer Institute. A431 cells were inoculated to the flank of male athymic mice (Harlan, 5-6 weeks of age) subcutaneously at 2.5 × 10^6^ cells per site in 100 μl PBS. Tumor growth as well as mouse body weight was closely monitored. We calculated tumor size using length x width^2^/2. Once tumor size reached 40-50 mm^3^ (approximately 4 days after A431 cell inoculation), the mice were treated with vehicle or EP (0.5 mg/kg) i.p. daily. Some of the EP-treated mice, once their tumor size reached approximately 400 mm^3^ (after 9 days of EP treatment), were treated with EP (0.5 mg/kg daily) plus hPEPD-G278D (4 mg/kg) which was given i.p. every other day. EP and hPEPD-G278D were prepared in PBS at 0.1 mg/ml and 0.8 mg/ml, respectively, and each agent was administered to a mouse in approximately 100 μl volume. When EP and hPEPD-G278D were both given to the same mice on the same day, EP was given 1 h earlier than hPEPD-G278D. Tumors grew exceedingly fast, and the mice treated by vehicle or EP only were killed after 13 days of treatment, as some of the tumors began to show sign of potential necrosis. Mice treated by EP plus hPEPD-G278D showed pronounced tumor regression and were killed after the 8^th^ dose of hPEPD-G278D, per IACUC guideline, as a high percentage of the tumors became necrotic at that time. All mice were killed 24 h after the final treatment; blood samples and tumors were collected from the mice when they were killed.

### Measurement of plasma mPEPD and hPEPD-G278D

Plasma levels of mPEPD and/or hPEPD-G278D were measured by ELISA as previously described [17].

### Statistical analysis

Analysis of variance was used for multi-group comparisons. P value of 0.05 or lower was considered statistically significant.

## SUPPLEMENTARY MATERIALS FIGURES AND TABLES


