# Supplementary material for: Dual inhibition of ErbB1 and ErbB2 in cancer by recombinant human prolidase mutant hPEPD-G278D
Source: Oncotarget. 2016 Jun 6;7(27):42340–52. doi: 10.18632/oncotarget.9851 (PMC5173139; doi:10.18632/oncotarget.9851)
Supplement: Supplementary file 1 [file oncotarget-07-42340-s001.pdf]

SUPPLEMENTARY MATERIALS

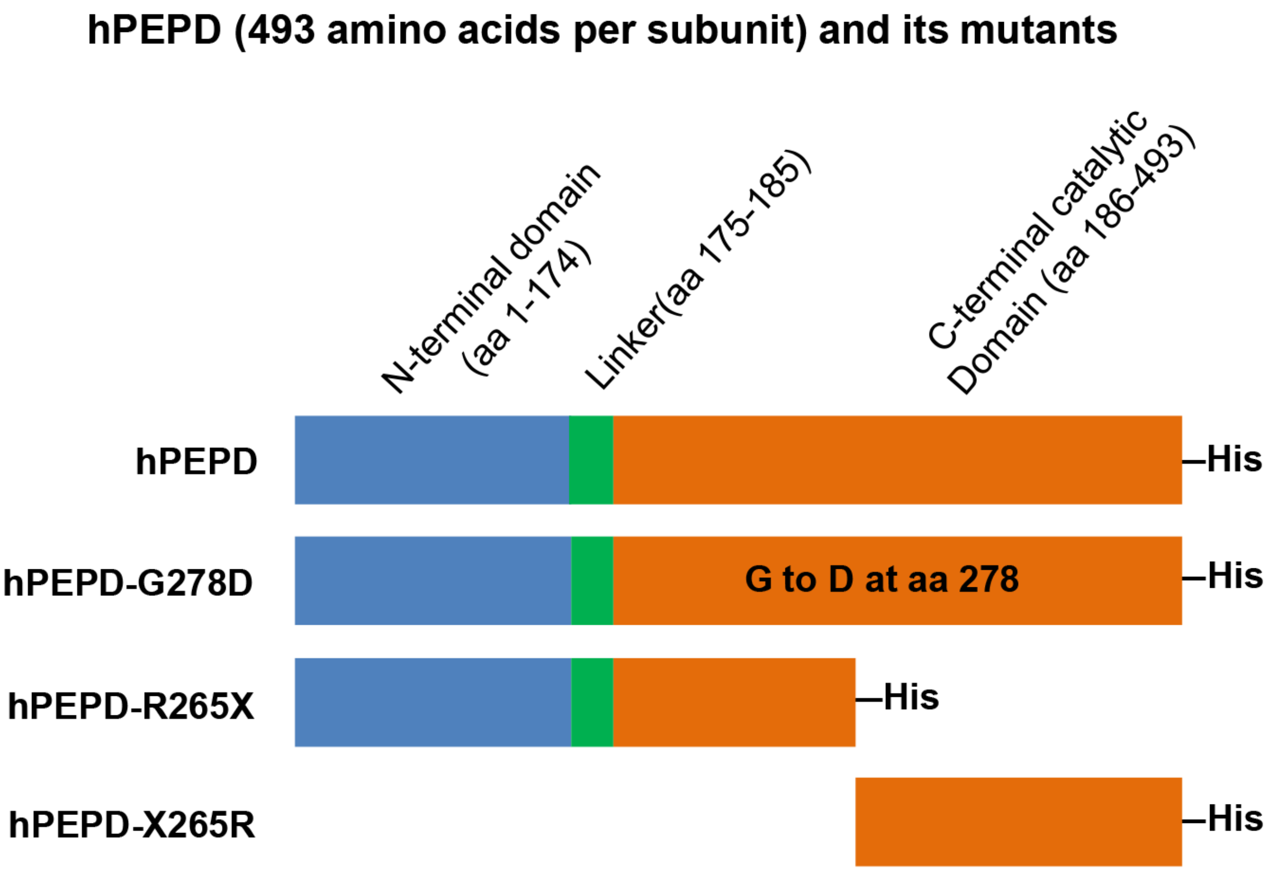

Supplementary Figure S1: Sequence information of hPEPD, hPEPD-G278D, hPEPD-R265X and hPEPD-X265R. Each protein has His tagged to its carboxy terminus.

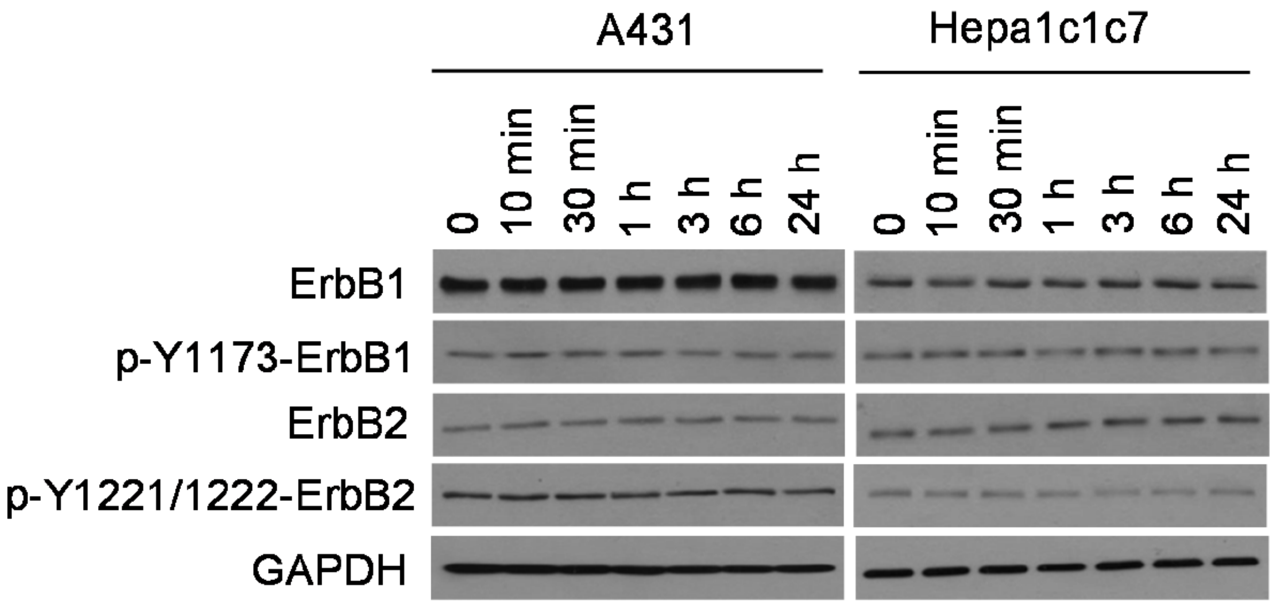

Supplementary Figure S2: No effect of solvent on ErbB1 and ErbB2 in cultured cells. A431 cells and Hepa1c1c7 cells were either untreated or treated with solvent (0.1% PBS in medium) for 10 min to 24 h, followed by preparation of cell lysates and measured of ErbB1 and ErbB2 and their phosphorylation status by IB. GAPDH was used as a loading control.

Supplementary Table S1: The sequences of primers used for cloning ERBB1 and generating deletion mutants of ERBB1

Targeting vector	Primer	Sequence
pCMV6-A-ERBB1-Puro	For	5'-GCGATCGCcggccccctgactccgtccag-3'
	Rev	5'-ACGCGTtcatgctccaataaattcactgctttgtggc-3'
pCMV6-XL5-ERBB1/delD1	For	5'-cggggagcagcgatggacatagtcagcagt-3'
	Rev	5'-actgctgactatgtccatcgctgctccccg-3'
pCMV6-XL5-ERBB1/delD2	For	5'-catccagtggcggtgtggggccgaca-3'
	Rev	5'-tgtcggccccacaccgccactggatg-3'
pCMV6-XL5-ERBB1/delD3	For	5'-cggaggtcccaaaggctcggacgcac-3'
	Rev	5'-gtgcgtccgagcctttgggacctccg-3'
pCMV6-XL5-ERBB1/delD4	For	5'-ccgtaggtgcagtttggatgcagtttttccagtttattg-3'
	Rev	5'-caataaactggaaaaaactgcatccaaactgcacctacgg-3'
